# Redesigning Isolation Practices: Evaluation of a Comprehensive Protocol for Respiratory Virus Control Including Cycle Threshold (Ct) Value Dynamics

**DOI:** 10.3390/v18010040

**Published:** 2025-12-25

**Authors:** Stefanie Lemmens, Kevin Janssen, Tine Nelis, Ahmed Elmahy, Noëlla Pierlet, Els Oris, Deborah Steensels

**Affiliations:** 1 Department of Molecular Microbiology, Ziekenhuis Oost-Limburg, 3600 Genk, Belgium; stefanie.lemmens@zol.be (S.L.); kevin.janssen@zol.be (K.J.); els.oris@zol.be (E.O.); 2 Department of Infection Prevention and Control, Ziekenhuis Oost-Limburg, 3600 Genk, Belgium; tine.nelis@zol.be; 3 Central Data Coordination, Hospital Outbreak Support Team, Ziekenhuis Oost-Limburg, 3600 Genk, Belgium; ahmed.elmahy@zol.be (A.E.); noella.pierlet@zol.be (N.P.); 4 Faculty of Medicine and Life Sciences—Biomed, University Hasselt, 3500 Hasselt, Belgium

**Keywords:** isolation practices, cycle threshold dynamics, SARS-CoV-2, non-SARS-CoV-2 viruses

## Abstract

The recent literature has debunked the widespread hypothesis that viruses are primarily transmitted via droplets and not beyond 1.5 m, and transmission via contact has been downplayed. Hence, an evidence-based revision of the existing isolation guidelines for respiratory viruses was needed. Therefore, a completely new protocol for respiratory virus isolation in terms of personal protective equipment and patient room air purification was evaluated. Isolation relief criteria based on Ct values in follow-up sampling were assessed. A Ct value of <28 was employed as a proxy for potential active replication and associated transmissibility. Between 25% and 50% of patients who tested positive for RSV, HRV, hMPV, or SARS-CoV-2 continued to exhibit high viral loads on day 7 post-initial diagnosis, underscoring the potential for sustained infectivity. Hence, the discontinuation of isolation measures for these patients without follow-up testing may carry a considerable risk of ongoing viral transmission. On the contrary, only 7% of patients positive for Flu and 14% for PIV had a follow-up sample on day 7 with a Ct value of less than 28. Ct values increased more rapidly in influenza, indicating faster viral clearance compared to other respiratory viruses. Based on these results, the policy of a standard 7-day isolation period without follow-up testing could be adopted for influenza-positive patients.

## 1. Introduction

In response to the SARS-CoV-2 pandemic, research on the transmission pathways of respiratory viruses was conducted worldwide, resulting in significant new insights. The recent literature has debunked the widespread hypothesis that viruses are primarily transmitted via droplets and not beyond 1.5 m [1]. A droplet quickly divides into small aerosols and spreads meters further through the air. Transmission via contact has also been downplayed. The risk of contracting a respiratory virus through contact with materials is much smaller than previously thought. These new scientific insights have not yet been incorporated into current national and international guidelines. And yet a revision of the existing isolation guidelines for respiratory viruses was deemed necessary, since the multitude of measures (various forms of isolation, isolation durations, and personal protective equipment (PPE) for different respiratory viruses) also led to confusion in the workplace.

Since October 2023, Ziekenhuis Oost-Limburg (tertiary center, 1081 beds) has implemented new isolation measures to prevent the nosocomial spread of respiratory viruses. The goal was to implement evidence-based policies, uniform for all respiratory viruses (KISS principle), to ensure the safety of healthcare workers and patients, using practically feasible measures.

Two major paradigm shifts were introduced, both in terms of personal protective equipment and the focus on the number of air changes per hour. Regarding personal protective equipment, the focus was placed on strict mask-wearing and eye protection while adhering to standard precautions including appropriate hand hygiene. The standard use of gloves and gowns was abandoned. Completely new was the attention to air purification, as we decided to use a mobile air purification device for each patient in respiratory virus isolation. This was to reduce the viral load in the room and to allow the patient’s room door to remain open. Several studies have shown the impact of increased ventilation rates on the extraction of pathogens from the indoor environment and a reduced risk of transmission [2,3,4,5]. Keeping the door open allows healthcare providers to monitor the patient more effectively while reducing the patient’s sense of isolation.

As the first part of this study, the Infection Prevention and Control Department evaluated the impact of the implemented changes on staff, patients, and nosocomial infection rates.

In addition, the isolation duration and criteria for isolation relieve were standardized. A minimum isolation duration of 7 days (counting from the start of symptoms or—when not clear—from the day of positive PCR result) was implemented for all admitted patients with a respiratory virus infection. For those still hospitalized, follow-up sampling on day 7 was introduced in order to evaluate the potential contagiousness and to make an informed decision regarding the end of isolation measures.

For SARS-CoV-2, Ct values of <28 are typically interpreted as indicating active viral replication and potential contagiousness, based on studies of viral culture and secondary infection rates [6,7,8,9]. However, replication efficiency can vary between variants and subvariants. Previous studies have reported differences in viral load and culture positivity across SARS-CoV-2 lineages. For instance, Sentis et al. observed lower viral loads in nasopharyngeal swabs following the emergence of Omicron variants. Conversely, other reports indicate that Ct values were generally higher for Omicron compared to pre-Omicron specimens, particularly during the first week of illness [10]. Fall et al. further demonstrated that infectious virus was recovered more frequently from Delta samples than from Omicron samples (78% vs. 61%, Fisher’s exact test, *p* = 0.0009), suggesting that the presence of infectious virus alone does not fully account for Omicron’s increased transmissibility [11]. It is important to note that transmission potential is also influenced by immune evasion following prior infection and/or vaccination.

Within Omicron subvariants, recovery of infectious virus was consistently associated with lower mean Ct values, regardless of lineage. For example, mean Ct values for culture-positive versus culture-negative samples were as follows: BA.1 (16.4 vs. 20.5, *p* < 0.0001), BA.1.1 (18.5 vs. 25.1, *p* = 0.004), BA.2 (20.3 vs. 24.9, *p* < 0.0001), BA.2.12.1 (21.2 vs. 23.7, *p* = 0.19), BA.4 (19.3 vs. 25.1, *p* = 0.0001), and BA.5 (18.5 vs. 26.4, *p* < 0.0001) [12]. These findings are consistent with data from Wooding et al., who reported no recovery of infectious virus above Ct 25 [13]. Data from the Belgian National Reference Center for Respiratory Viruses, collected throughout the COVID-19 pandemic, similarly indicate that samples with Ct values > 26 were uniformly negative in viral culture (unpublished data). Based on these data, the selected cut-off of Ct > 28 to define the end of contagiousness appears to be a conservative and appropriate choice.

Little data is available regarding viral replication dynamics for influenza, RSV, and other non-SARS-CoV-2 pathogens and how they correlate with stage of infection or contagiousness. A study by Ehrenzeller et al. has already shown that Ct values relative to symptom onset for influenza, RSV, and other non-SARS-CoV-2 respiratory viruses generally mirror patterns seen with SARS-CoV-2 [14]. However, they point out that further data on associations between Ct values and viral viability, transmissibility, host characteristics, and response to treatment for non-SARS-CoV-2 respiratory viruses are needed to determine how clinicians and infection prevention specialists might integrate Ct values into treatment and isolation decisions.

As the second part of this study, we evaluated the Ct value dynamics in systematic follow-up samples of both non-SARS-CoV-2 pathogens and SARS-CoV-2 during the respiratory seasons of 2023–2025. The effect of follow-up testing after 7 days of isolation on isolation duration and the number of hospital days was also assessed. For influenza, we correlated Ct value with treatment response to oseltamivir.

## 2. Materials and Methods

### 2.1. Evaluation of the Revised Respiratory Isolation Measures

During the first three months following the implementation of the new policy, on-site inspections were carried out by an infection prevention nurse for every patient placed in respiratory virus isolation. Nursing staff (and occasionally the attending physician) were interviewed regarding their experience with the revised policy and were provided with guidance when necessary. Additionally, a sample of patients was surveyed about their experience with the air purification device. All feedback was recorded in writing and subsequently categorized for analysis. This evaluation aimed to ensure adherence to the new policy and to identify opportunities for improvement. In addition, the incidence of nosocomial infections and outbreaks was compared to that of the preceding autumn–winter seasons.

### 2.2. Clinical Specimens and Patients

In this retrospective observational study, patients with a respiratory infection were tested using combined naso- and oropharyngeal swabs. The study was approved by CTU ZOL Genk (study number: Z-2025055).

Between 1 October 2023 and 31 March 2025, patients who underwent follow-up sampling 4–9 days after the first sample were included. Ct values of day 1 and day 7 and further follow-up samples (if applicable) were registered.

**Figure 1:**
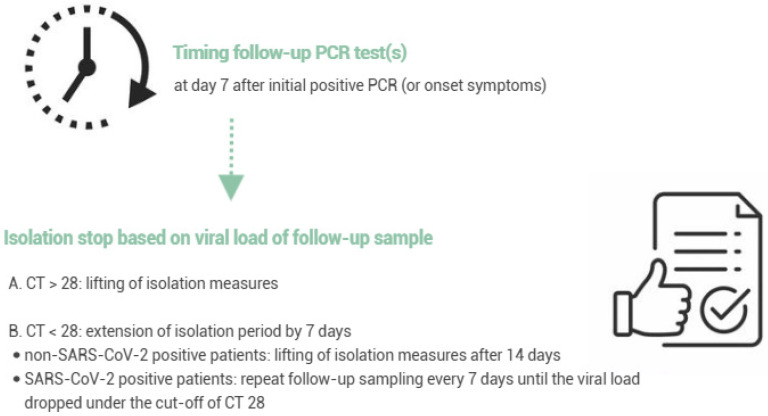
Overview of the patient isolation policy based on the viral load of follow-up PCR testing.

A follow-up test was performed 7 days after the onset of symptoms or a positive test if the patient was still hospitalized. The hospital policy adopted a cut-off of Ct value ≥ 28 in order to relieve isolation measures. When the follow-up sample tested positive with a Ct value < 28, the patient was isolated for 7 extra days. Patients positive for non-SARS-CoV-2 respiratory viruses could leave isolation after 14 days. For SARS-CoV-2-positive patients, follow-up sampling was repeated every 7 days until the viral load dropped under a Ct value of 28 (~5.83 log10 copies/mL) (Figure 1).

### 2.3. Molecular Assays

Samples were tested for semiquantitative detection and differentiation of respiratory viruses using two multiplex PCR assays, Xpert^®^ Xpress CoV-2/Flu/RSV plus (Cepheid Inc., Sunnyvale, CA, USA) and/or Allplex™ RV Master Assay (Seegene Inc., Seoul, Republic of Korea). The GeneXpert triplex test was used for patients in the emergency department and the Allplex RV Master assay for in-hospital patients. The GeneXpert triplex test is able to target SARS-CoV-2, influenza A, influenza B, and RSV. The RV Master panel includes the following targets: SARS-CoV-2, influenza, RSV, hMPV, parainfluenza virus 1–4, adenovirus, and rhinovirus. Both assays were performed according to the manufacturers’ instructions.

### 2.4. Associations Between Treatment and Viral Loads

For SARS-CoV-2, RSV, hMPV, parainfluenza virus 1–4, adenovirus, and rhinovirus, no antiviral therapy was administered during the study period.

According to local antibiotic stewardship guidelines, all patients hospitalized with influenza virus infection should receive oseltamivir at a dosage of 75 mg twice daily for adults and children weighing more than 40 kg (with dose adjustments for renal impairment and for children weighing less than 40 kg). Treatment should ideally be initiated within 48 h of symptom onset and is typically administered for 5 days, with extension to 10 days in immunocompromised patients.

The initiation date and duration of oseltamivir treatment were extracted for all included influenza patients. Patients without treatment were indicated as “no treatment found.” The Ct values of oseltamivir-treated and untreated patients were compared on day 1 and day 7 using the Kruskal–Wallis test followed by Dunn’s multiple comparisons test, as well as nonlinear regression analysis. Additionally, a sample size calculation was performed to determine whether the number of patients per group provided 80% power to detect a difference of 3 Ct values between the groups.

### 2.5. Statistical Analyses

Data management was performed using GraphPad Prism version 10.4.1. (GraphPad Software, La Jolla, CA, USA). Statistical comparisons between multiple groups were performed using the Kruskal–Wallis test followed by Dunn’s multiple comparisons test. To evaluate differences in regression slopes and determine whether they were statistically identical, nonlinear regression analysis was applied. Associations between categorical variables were assessed using Fisher’s exact test. A *p*-value < 0.05 was considered significant.

## 3. Results

### 3.1. Evaluation of the Revised Respiratory Isolation Measures

The new policy was received very positively by staff and doctors. Healthcare workers reported a considerably decreased workload due to the adjustment of PPE, and patients reported feeling less lonely. Staff also reported feeling safer due to improved air quality. The distribution flow for the air purification devices was adjusted several times to achieve a balance between delivery speed and logistical feasibility. Compared to the previous autumn–winter seasons, the new policy did not lead to an increase in nosocomial infections or outbreaks, suggesting that the relaxation of contact isolation measures (abandoning gloves and gowns) combined with increased air purification is effective and safe. 

### 3.2. Patient Characteristics

Between 1 October 2023 and 31 March 2025, a total of 977 follow-up samples were taken from 913 hospitalized patients. Patient characteristics are shown in Table 1.

In total, 379 patients were positive for SARS-CoV-2 on day 1 and were still hospitalized after 7 days. In the non-SARS-CoV-2 group, 535 patients in total were positive for the different viruses on day 1 and still hospitalized by day 7 (for details per virus, see Table 1). The population studied had a median age of 80 years. However, pediatric cases were also represented in the cohort. The gender distribution was 49% female and 51% male. Information regarding the hospitalization department was also obtained. Most patients were hospitalized in the geriatrics department.

Table 2 gives an overview of the positivity rate of follow-up samples per respiratory virus.

**Figure 2:**
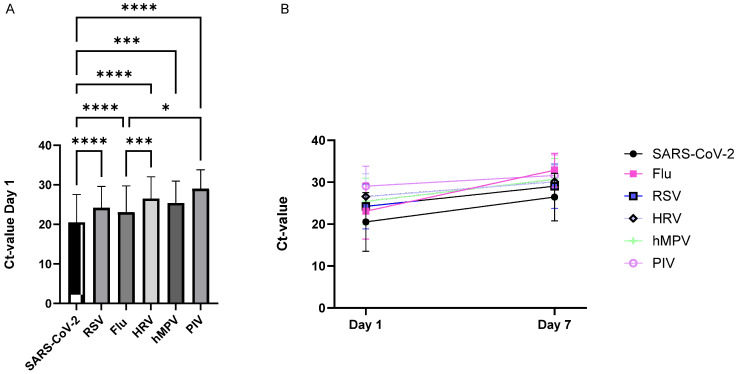
Comparison of the Ct values of day 1 and day 7 samples positive for SARS-CoV-2 and for non-SARS-CoV-2 pathogens. (**A**) The mean Ct value of SARS-CoV-2 on day 1 was significantly lower compared to that of all other respiratory viruses (* *p* < 0.05, *** *p* < 0.001, **** *p* < 0.0001; Kruskal–Wallis test). (**B**) The Ct values of influenza increased faster compared to the Ct values of the other respiratory viruses (nonlinear fit).

The Ct values of day 1 samples positive for SARS-CoV-2 and for non-SARS-CoV-2 pathogens were compared. The mean Ct value of SARS-CoV-2 on day 1 was significantly lower compared to that of all other respiratory viruses, suggesting higher contagiousness for SARS-CoV-2-infected patients at the onset of symptoms or admission to the emergency department (Figure 2).

### 3.3. Ct Value Dynamics

**Figure 3:**
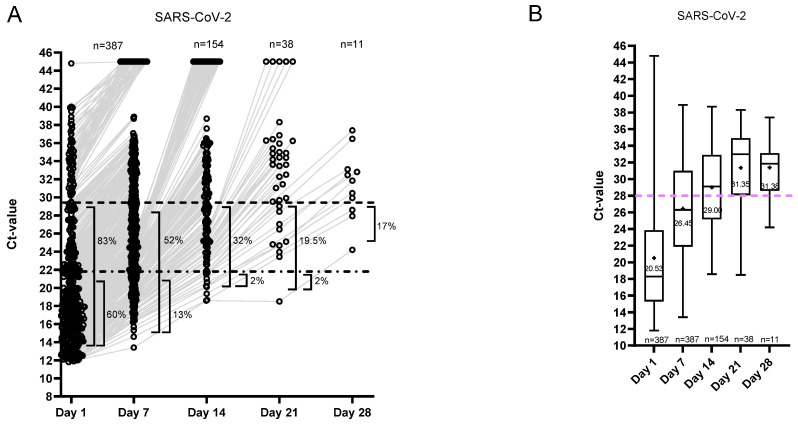
Ct value dynamics for SARS-CoV-2. (**A**) Distribution of Ct values and the percentages of positive samples per sampling day. The samples resulting in ‘not detected’ are shown as Ct 45. (**B**) The median and range of the Ct values of the positive samples. The plus indicates the mean Ct value per day.

Ct value dynamics for the SARS-CoV-2 group are shown in Figure 3. On day 1, 83% of the patients were positive with Ct < 28, and 60% of the patients had Ct < 20 (Figure 3A). An overview of the percentage of samples relative to Ct values of 28, 25, and 20 per sampling day is shown in Supplementary Figure S1.

On day 7, 52% of the SARS-CoV-2-positive patients were positive with Ct < 28 (13% with Ct < 20), and on day 14, 32% were still positive with Ct < 28 (2% with Ct < 20) (Figure 3A). The mean Ct value of the SARS-CoV-2 group exceeded Ct ≥ 28 in the second follow-up sample on day 14 (Figure 3B).

**Figure 4:**
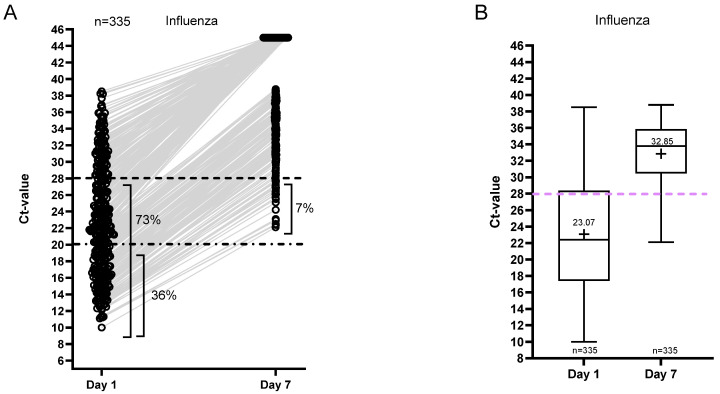
Ct value dynamics for influenza. (**A**) Distribution of Ct values and the percentages of positive samples per sampling day. The samples resulting in ‘not detected’ are shown as Ct 45. (**B**) Median and range of the Ct values of the positive samples. The plus indicates the mean Ct value per day.

In the influenza virus group, on the other hand, only 7% remained positive with Ct < 28 on day 7, and no follow-up samples were positive with a Ct value < 20. The mean Ct value of this group on day 7 was 33 (Figure 4). Moreover, linear regression of the Ct values on day 1 and day 7 was fitted for the different viruses, and slopes were compared. Figure 2B shows that the Ct values of influenza increase faster compared to the Ct values of the other respiratory viruses.

**Figure 5:**
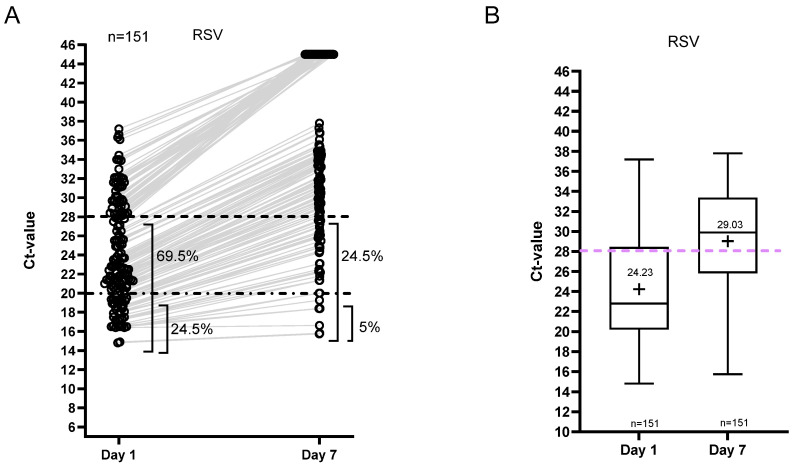
Ct value dynamics for RSV. (**A**) Distribution of Ct values and the percentages of positive samples per sampling day. The samples resulting in ‘not detected’ are shown as Ct 45. (**B**) The median and range of the Ct values of the positive samples. The plus indicates the mean Ct value per day.

For the RSV group, Ct value dynamics are shown in Figure 5. On day 7, 25% of patients remained positive with Ct < 28, whereas 5% had Ct < 20 (15% had Ct < 25; Figure S1). The mean Ct value of the RSV group on day 7 was 29 (Figure 5B).

**Figure 6:**
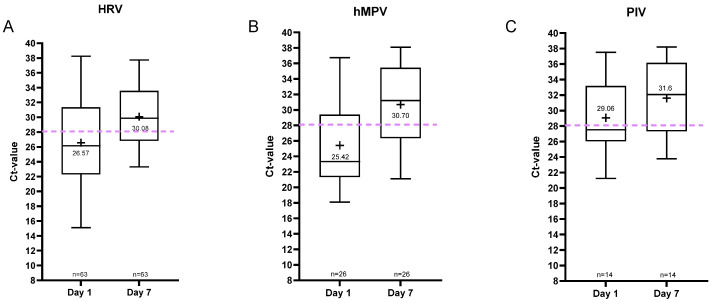
Median and range of the Ct values of the positive samples for (**A**) HRV, (**B**) hMPV, and (**C**) PIV per sampling day. The plus indicates the mean Ct value per day.

The patient numbers were much smaller for the rhinovirus (n = 63), human metapneumovirus (n = 26), and parainfluenza virus (n = 14) groups. Data are shown in Figure 6 and Supplementary Figure S2. For these viruses, the mean Ct value on day 7 was ≥30 (Figure 6). Furthermore, comparison of the proportion of patients with a Ct value < 28 on day 7 across different viral infections reveals that a significantly higher percentage of SARS-CoV-2 patients (52%; Fisher’s exact test: *p* < 0.0001) retained a Ct value < 28, in contrast to markedly fewer patients in the influenza (7%; Fisher’s exact test: *p* < 0.0001) and parainfluenza virus (PIV) groups (14%; Fisher’s exact test: *p* = 0.000516).

### 3.4. Isolation and Hospitalization Duration

**Figure 7:**
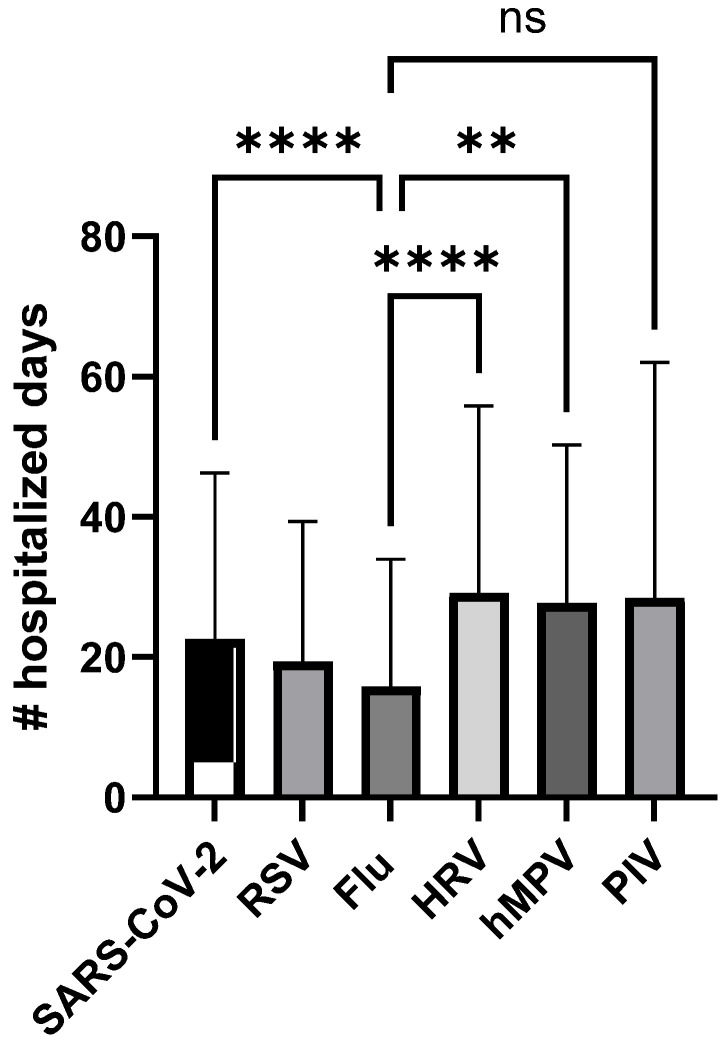
Total duration of hospitalization for the different patient groups (** *p* < 0.001, **** *p* < 0.0001; ns, non significant; Kruskal–Wallis test).

The total duration of hospitalization for the different patient groups is shown in Figure 7. The number of hospitalized days was significantly lower for Flu patients (mean = 15.9 days; *p* < 0.01) compared to SARS-CoV-2 (mean = 22.58 days), HRV (mean = 29.16 days), and hMPV (mean = 27.72 days). The number of isolation days was significantly higher for SARS-CoV-2 patients (mean = 9.6 days; *p* < 0.001) compared to non-SARS-CoV-2 patients (5.4–7.8 days), representing the isolation policy.

### 3.5. Associations Between Oseltamivir Treatment and Viral Loads for Influenza Patients

**Figure 8:**
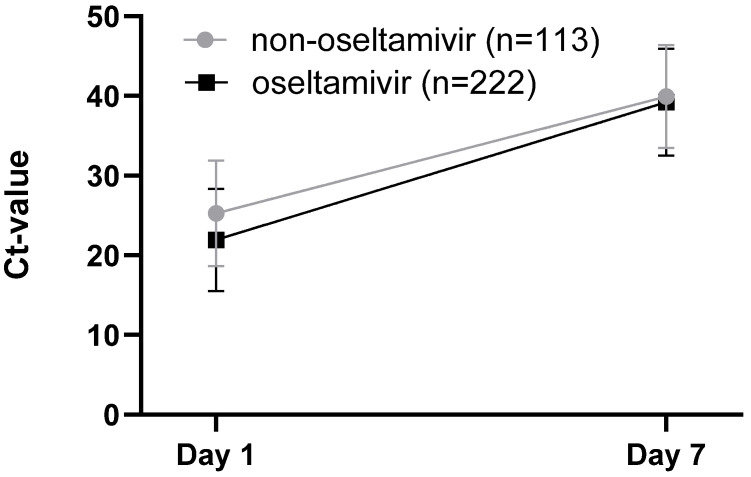
Association between oseltamivir treatment and Ct value dynamics.

The association between oseltamivir treatment and Ct value dynamics was assessed. No differences in Ct values on day 7 between the oseltamivir-treated and non-treated groups were observed (Figure 8). A sample size calculation was performed to determine whether the number of patients per group was sufficient to have a power of 80% to detect a difference of 3 Ct-values between the groups. Considering a standard deviation of 6.7 Ct and a significance level of 0.05, a sample size of n = 80 (n = 113 for the non-oseltamivir and n = 222 for the non-oseltamivir group) should be sufficient to detect group differences.

## 4. Discussion

The introduction of a completely new protocol for respiratory virus isolation required thorough preparation. All stakeholders were actively involved in the process, such as hospital staff, technical departments, the supply service, and head nurses. Adequate training and education contributed to a smooth transition. In the initial months following implementation, the Infection Prevention and Control team conducted intensive follow-up. The revised isolation policy was well accepted, reduced staff workload, improved perceived air quality, and did not increase nosocomial infection rates, supporting its safety and effectiveness.

In order to evaluate our new criteria for isolation relief, we studied Ct value dynamics for respiratory viruses from symptomatic patients, with follow-up sampling on day 7 for all viruses and sampling every 7 days until the viral load dropped ≥ Ct 28 for SARS-CoV-2. A Ct value of <28 was employed as a proxy for potential active replication and associated transmissibility, based on studies of viral culture and secondary infection rates [6,7,8,9].

Our data suggest that the Ct value dynamics for SARS-CoV-2 and non-SARS-CoV-2 viruses are different. Firstly, the Ct values for SARS-CoV-2 were significantly lower in the day 1 sample, indicative of a higher viral load at the time of initial testing. SARS-CoV-2 viral load is known to peak around symptom onset, while influenza, RSV, HRV, and hMPV typically peak after symptom onset [15,16,17,18]. As the timing of diagnostic testing relative to symptom onset was not standardized across patients in this study, no definitive conclusions can be drawn from this observation.

In addition, for the SARS-CoV-2 patient group, the mean Ct value exceeded the cut-off of ≥28 only by day 14 following initial sampling, whereas in the patient groups for the other viruses, this threshold was already reached by day 7. A comparison of the proportion of patients with a Ct value < 28 on day 7 indicates that a significantly higher number of SARS-CoV-2 patients still exhibited low Ct values, suggesting ongoing viral replication. Moreover, between 25% and 50% of patients who tested positive for RSV, hRV, hMPV, or SARS-CoV-2 continued to exhibit high viral loads on day 7 post-initial diagnosis, underscoring the possibility of sustained infectivity. Hence, the discontinuation of isolation measures for these patient groups without follow-up testing may carry a considerable risk of ongoing viral transmission.

The efficacy of the use of Ct values for isolation policy and decision making has previously been described. It has been shown that the efficacy of ending isolation based on Ct values could improve bed utilization without increasing the risk of transmission among patients with COVID-19 requiring therapy for >20 days after symptom onset [19]. This is supported by the findings of another study, where, by day 21 following initial test positivity, all 14 samples collected from seven patients exhibited Ct values above 30 (mean Ct: 31.6), suggesting a reduced likelihood of infectivity [20]. Furthermore, no cases of nosocomial transmission were linked to patients who were released from isolation on day 21 within the healthcare system studied. Moreover, a systematic review and meta-analysis reported that SARS-CoV-2 RNA shedding in the upper respiratory tract had a mean duration of 17.0 days [21], which could also explain the reduced transmission risk 21 days after symptom onset.

In our cohort of SARS-CoV-2-positive patients, nearly one-third continued to exhibit high viral loads on day 14 following initial testing. Given that the interquartile range (IQR) of Ct values exceeded 28 on both day 21 and day 28, these findings support the consideration of discontinuing follow-up testing from day 14 onward and releasing patients from isolation on day 21 if the viral load has not decreased sufficiently by day 14.

A similar pattern of viral loads and Ct value dynamics has been described by Erhenzeller and colleagues. They studied non-SARS-CoV-2 viruses (influenza, RSV, PIV, HRV, and hMPV) and found that 7 to 9 days from symptom onset were needed for the Ct value to exceed ≥28 [14].

On the contrary, only 7% of patients positive for Flu and 14% for PIV had a follow-up sample on day 7 with a Ct value of less than 28. This was significantly lower compared to the percentage of patients with Ct < 28 on day 7 in the other respiratory virus groups.

Linear regression analysis of Ct values on day 1 and day 7 revealed a more rapid increase in Ct values for influenza, indicating faster viral clearance compared to the other respiratory viruses. This observation aligns with previous findings in healthy adults, where influenza viral load typically declines rapidly within approximately 5 to 7 days post-symptom onset [22].

The shorter duration of hospitalization combined with the low proportion of patients who remained positive on day 7 suggests that influenza infections may resolve more rapidly compared to other respiratory viral infections. Determining the acceptable proportion of patients who continue to exhibit high viral loads on day 7 post-diagnosis, in order to justify the discontinuation of follow-up testing, remains a complex and clinically challenging decision. Based on the findings of this study, however, the Infection Prevention and Control department will adopt the policy of a standard 7-day isolation period without follow-up testing for influenza-positive patients.

The association between oseltamivir treatment and Ct value dynamics in Flu-positive patients has also been assessed. No differences in Ct values were detected between the treatment and non-treatment groups. However, it was described that patients treated with oseltamivir within 4 days of symptom onset had a significantly higher likelihood of viral RNA clearance by day 7 compared to untreated patients [23,24,25,26].

### Limitations of This Study

Firstly, the Ct value is not a perfect measure of viral burden; it can vary depending on sample site, sample quality, assay type, and assay sensitivity and may not have a linear relationship with analyte quantity [27,28,29]. Nonetheless, it gives an approximate, semiquantitative measure of viral burden, particularly when serial samples with sequential Ct values are available [6,30,31]. A strong relationship between Ct values, viral culture, and contagiousness has been observed for SARS-CoV-2 [32,33]. For the non-SARS-CoV-2 viruses, the general pattern of low Ct values near symptom onset followed by a steady increase over the ensuing days with substantial patient-to-patient variability broadly mirrors the viral dynamics seen with the SARS-CoV-2 Omicron variant [14]. The authors concluded that the parallels between Ct dynamics for SARS-CoV-2 and other respiratory viruses are helpful insofar as many clinicians and infection control practitioners have learned how to integrate Ct values into their decision making for patients with positive SARS-CoV-2 tests with regard to the necessity and duration of isolation, amongst other factors.

Also, the interpretation of SARS-CoV-2 Ct values is guided by viral culture studies correlating Ct values with the probability of recovering a culture-viable virus and by studies documenting a correlation between patients’ Ct values and the frequency of secondary transmission [8,29,30,34]. However, little data is available that correlates non-SARS-CoV-2 respiratory virus Ct values with viral culture or secondary transmission. One study showed that household transmission of influenza was correlated with Ct values < 30 [7]. Household transmission of influenza was 7-fold higher from people with Ct values < 30 vs. ≥30. For rhinovirus, generally high and steady Ct values suggest that the RNA of the virus is present but that the virus is not replicating, possibly indicating colonization rather than acute infection [35].

Other limitations of our study include variable and, for some viruses, small sample sizes. The Ct values for SARS-CoV-2, RSV, and Flu were also generated with different molecular assays depending the patients location [emergency department vs. in-patient unit]. The timing of diagnostic testing relative to symptom onset was not standardized across patients. Hence, on day 7, patients might have been in a different phase of the infection, potentially leading to patient-level variations in viral dynamics. We were also not able to control confounders like the adequacy of sampling.

Lastly, the immune status of patients was not assessed in this study. However, we analyzed patient age, sex, and department of admission and found no significant differences among the various respiratory virus groups. Although certain departments (e.g., oncology) typically admit a higher proportion of immunocompromised patients, these departments were not disproportionately represented in any specific respiratory virus group compared to others.

## 5. Conclusions

A completely new protocol for respiratory virus isolation in terms of personal protective equipment and patient room air purification was described and evaluated. Isolation relief criteria based on Ct values in follow-up sampling were assessed. A Ct value of <28 was employed as a proxy for potential active replication and associated transmissibility. Between 25% and 50% of patients who tested positive for RSV, HRV, hMPV, or SARS-CoV-2 continued to exhibit high viral loads on day 7 post-initial diagnosis, underscoring the potential for sustained infectivity. Hence, the discontinuation of isolation measures for these patients without follow-up testing may carry a considerable risk of ongoing viral transmission. Nearly one-third of SARS-CoV-2 patients still had high viral loads by day 14. Our findings support discontinuing follow-up testing after day 14 and ending isolation on day 21 if the viral load remains high.

On the contrary, only 7% of patients positive for Flu and 14% for PIV had a follow-up sample on day 7 with a Ct value of less than 28. Ct values increased more rapidly in influenza, indicating faster viral clearance compared to other respiratory viruses. Based on these results, the policy of a standard 7-day isolation period without follow-up testing could be adopted for influenza-positive patients.

## Figures and Tables

**Table 1:** Demographic characteristics and hospital department information of patients. Internal medicine (INT) includes cardiology, endoscopy, gynecology, hematology, pulmonology, neurology, and oncology. Other departments are shown in the diverse category (pediatrics and neonatology, orthopedics, rehabilitation, psychiatry department, vascular diseases). Abbreviations: DEP: department; GER: geriatrics department; ICA: intensive care unit; INT: internal medicine; DIV: diverse departments.

RESPIRATORY VIRUS	TOTAL N. PATIENTS	N. FEMALES (%)	N. MALES (%)	MEDIAN AGE (95% CI)	DEP.	N. PATIENTS /DEPARTMENT
SARS-CoV-2	379	170 (45)	209 (65)	81 (80–82)	GER	237
					ICA	43
					INT	66
					DIV	33
FLU	321	161 (50)	160 (50)	79 (77–81)	GER	152
					ICA	46
					INT	94
					DIV	22
RSV	126	74 (59)	52 (41)	83 (80–86)	GER	74
					ICA	16
					REV	4
					INT	26
					DIV	10
HRV	52	25 (48)	27 (52)	79 (74–84)	GER	28
					ICA	9
					INT	11
					DIV	4
HMPV	23	9 (39)	14 (61)	77 (71–83)	GER	13
					ICA	4
					INT	4
					DIV	2
PIV	12	6 (50)	6 (50)	82 (68–95)	GER	7
					ICA	2
					INT	2
					DIV	1
ADV	1	0	1 (100)	0	DIV	1
TOTAL	913	445 (49)	468 (51)	80 (79–81)		

**Table 2:** Overview of the positivity rate of follow-up (FU) samples per respiratory virus.

RESPIRATORY VIRUS	FU SAMPLING	N. SAMPLES	N. POSITIVE SAMPLES (%)
SARS-CoV-2	Day 7	387	339 (88)
	Day 14	154	121 (79)
	Day 21	38	33 (87)
	Day 28	11	11 (100)
FLU	Day 7	335	153 (46)
RSV	Day 7	151	101 (67)
HRV	Day 7	63	49 (78)
HMPV	Day 7	26	20 (77)
PIV	Day 7	14	8 (57)
ADV	Day 7	1	1 (100)

## Data Availability

The data presented in this study are available on request from the corresponding author due to privacy reasons.

## Supplementary Materials

The following supporting information can be downloaded at https://www.mdpi.com/article/10.3390/v18010040/s1: Figure S1: Overview of the percentage of samples relative to Ct 28, 25, and 20 per sampling day for SARS-CoV-2, Flu, and RSV; Figure S2: Overview of the percentage of samples relative to Ct 28, 25, and 20 per sampling day for HRV, hMPV, and PIV.

## Abbreviations

The following abbreviations are used in this manuscript:

Ct	Cycle threshold
D	Days
DEP	Department
DIV	Diverse departments
FU	Follow up
GER	Geriatrics
ICA	Intensive care unit
INT	Internal medicine
N	Number
PCR	Polymerase chain reaction
PPE	Personal protective equipment
