# Supplementary material for: Redesigning Isolation Practices: Evaluation of a Comprehensive Protocol for Respiratory Virus Control Including Cycle Threshold (Ct) Value Dynamics
Source: Viruses. 2025 Dec 25;18(1):40. doi: 10.3390/v18010040 (PMC12846390; doi:10.3390/v18010040)
Supplement: Supplementary file 1 [file viruses-18-00040-s001.zip › viruses-4016935-supplementary.pdf]

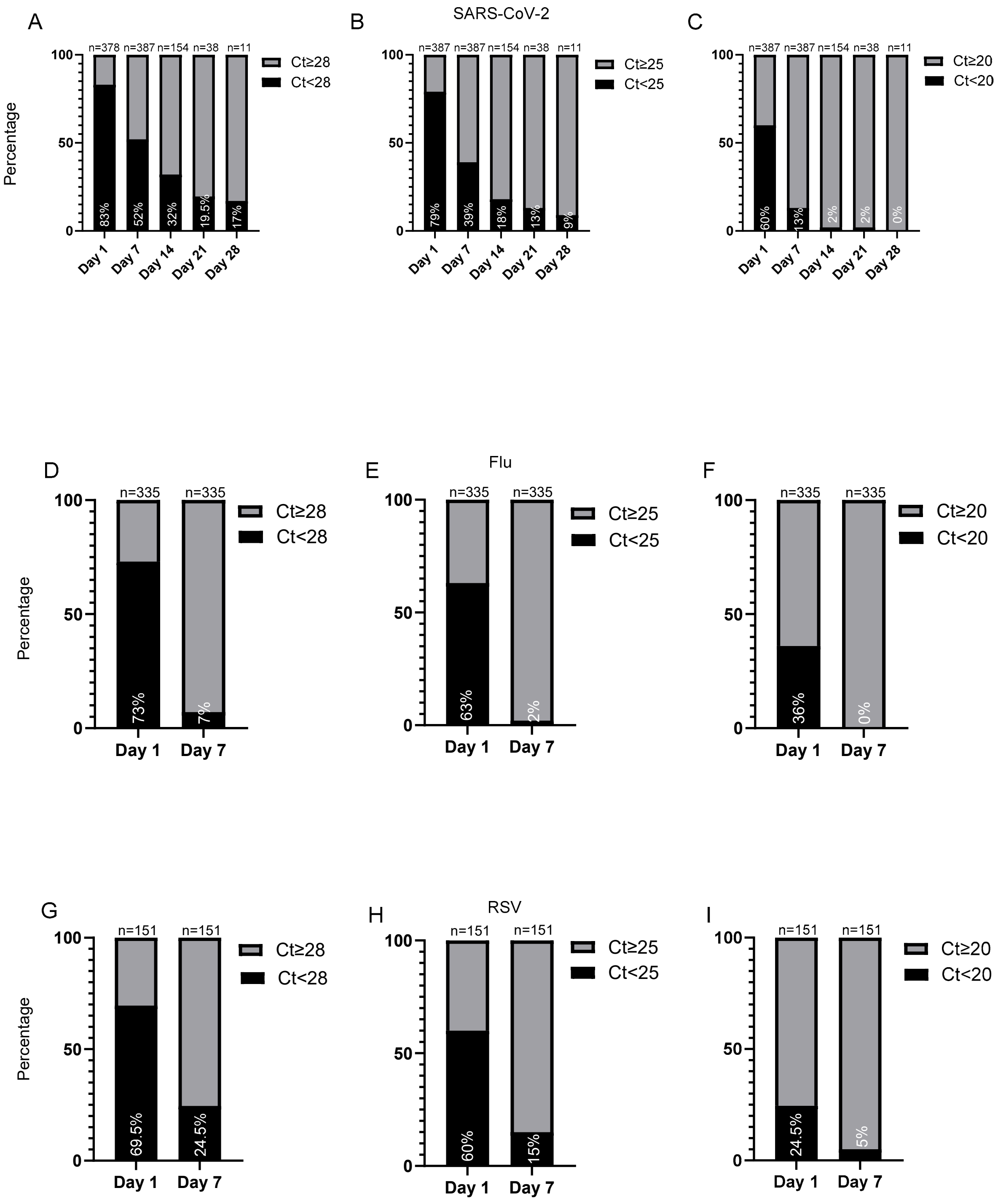

Supplementary Figure S1: Overview of the percentage of samples relative to Ct 28, 25 and 20 per sampling day for SARS-CoV-2, Flu and RSV.

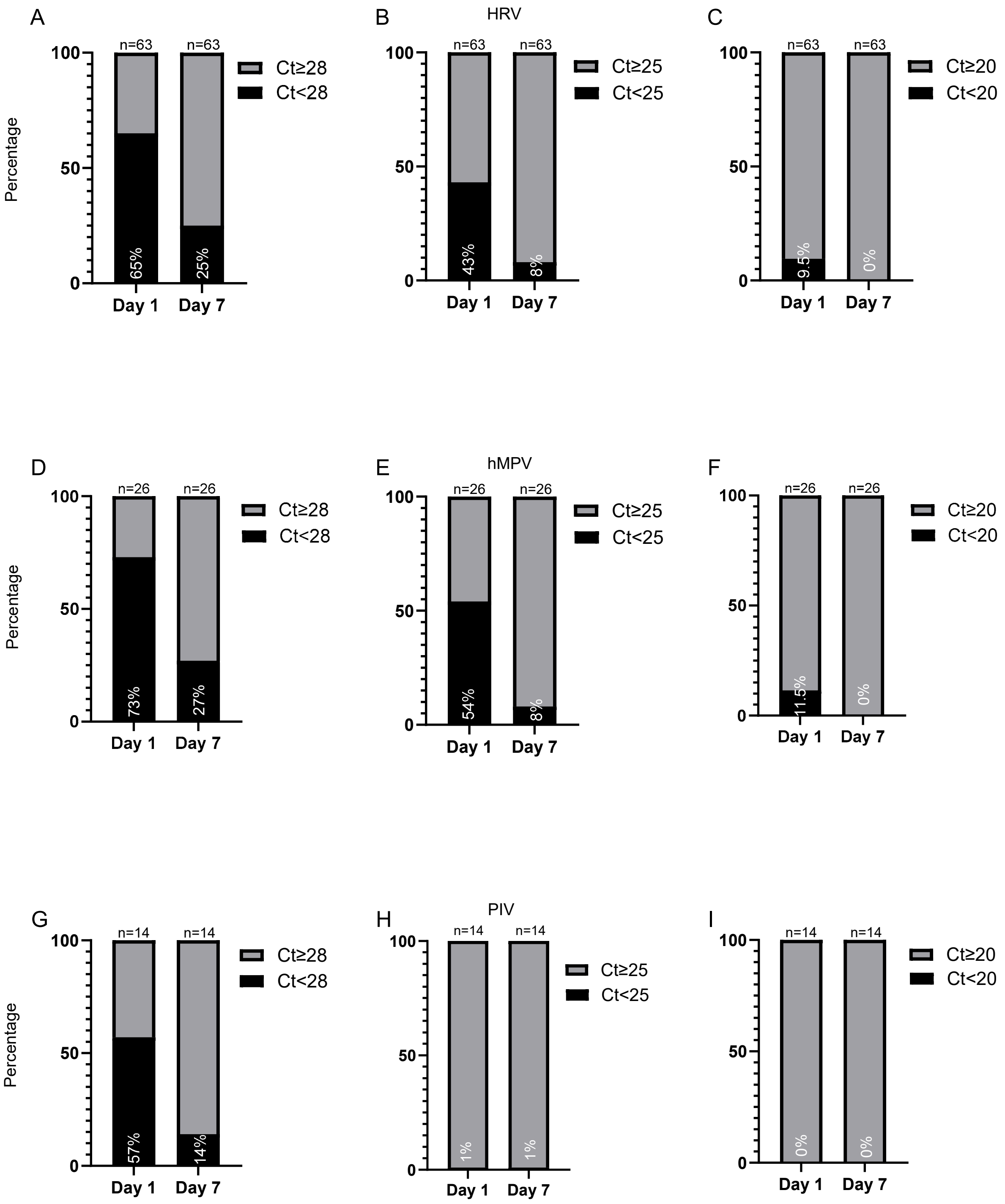

Supplementary Figure S2: Overview of the percentage of samples relative to Ct 28, 25 and 20 per sampling day for HRV, hMPV and PIV.
